# Supplementary material for: The effect of induced hyperammonaemia on sleep and melanopsin-mediated pupillary light response in patients with liver cirrhosis: A single-blinded randomized crossover trial
Source: PLoS One. 2022 Sep 28;17(9):e0275067. doi: 10.1371/journal.pone.0275067 (PMC9518847; doi:10.1371/journal.pone.0275067)
Supplement: S3 File — (PDF) [file pone.0275067.s003.pdf]

Project title:

The role of retinal blue light sensitivity in sleep disturbances associated with cirrhosis

Resume

Sleep disturbances are common among patients with cirrhosis. In this project we want to evaluate a potential pathophysiological mechanism. We will study retinal blue light reactivity and the continuous reaction time, before and after an oral amino acid challenge in patients with cirrhosis and healthy controls.

Project description:

In Denmark 12-15,000 patients currently suffer from cirrhosis (1, 2). It is known that approximately 50% of these patients will experience sleep disturbances such as difficulties falling asleep, nightly awakenings and abnormal daytime sleepiness (3). The sleep disturbances are worsened during episodes of hepatic encephalopathy (HE), which is a condition characterized by altered level of consciousness and confusion secondary to liver insufficiency. Reduced liver function can lead to a high plasma level of ammonium which is believed to be essential in the pathogenesis of HE (4). It has been demonstrated that the degree of sleepiness increases in parallel with the plasma level of ammonia in both healthy individuals and patients with cirrhosis after ingestion of an oral amino acids challenge (5). The development of sleep disturbances in patients with cirrhosis involves alterations in the circadian sleep regulation, where the release of melatonin from the pineal gland has been observed to be delayed (3, 6). Furthermore, a reduction in the light induced melatonin suppression is seen (6) which brought together gives a firm indication of abnormal function of the retino-hypothalamic axis, although the exact mechanism is not well understood.

The circadian rhythm is in part regulated by light stimulation of retina, where photosensitive and melanopsin-containing ganglion cells send signals to the circadian pacemaker cells in hypothalamus (7). Melanopsin is a photopigment with a maximal sensitivity to light with a wavelength of 480 nm, corresponding to blue light. Melanopsin is found in non-image forming retinal ganglion cells (8). In addition to contributing to the regulation of the circadian rhythm these ganglion cells are also involved in the pupillary light reflex and their function can be studied by pupillometry, i.e. measurement of the pupillary contraction following retinal exposure to blue light (9).

The main hypothesis of this project is that in patients with cirrhosis and sleep disturbances the retina-hypothalamic axis is affected through abnormal function of the melanopsin-containing ganglion cells, measured by pupillometry. Furthermore, we expect the degree of abnormal pupillary response to blue light to correlate with the arterial ammonium concentration as well as the degree of hepatic encephalopathy. We will evaluate this hypothesis by studying sleep patterns (using an ActiGraph Sleep Monitoring System and standardized sleep questionnaires (Pittsburgh Sleep Quality Index and Karolinska Sleepiness Scale)), measurement of capillary and arterial ammonia, measurement of capillary adenosine, psychometric test (continuous reaction time and portosystemic encephalopathy syndrom test) and pupillometry in both healthy subjects and patients with cirrhosis before and after an oral amino acid challenge (AAC). We expect the AAC will induce hyperammonemia, sleep abnormalities and altered pupillometry in the healthy subjects in a mild degree, and even more so in patients with cirrhosis.

Subjects

- 16 healthy individuals
- 16 cirrhotic patients independent of etiology (Child-Pugh class A or B) from the Department of Hepatology, Rigshospitalet or the Gastro Unit, medical division, Hvidovre Hospital.

In order to evaluate the degree of cirrhosis, information from patient records will be used for calculating the Child-Pugh score. Concretely, already analyzed blood test results and observations of either ascites or encephalopathy reported in the records will be registered by the principal investigator. Patients will be recruited during hospital stays or visits in the out-patient clinic. Healthy subjects will be recruited among colleagues by the use of posters in the department of the principal investigator.

Randomization

The subjects will be randomized to either receiving the AAC or an isocaloric glucose solution at the first experimental day and the opposite at the second experimental day. The randomization is done by drawing of an envelope from containing an A4 piece of paper with either the text “1: AAC. 2: Glucose solution” or “1: Glucose solution. 2: AAC”. Eight identical envelopes from each category

will be created for each study group (healthy subjects and patients with cirrhosis) before initiation of the project. The study is not blinded.

Exclusion criteria

- Lack or withdrawal of informed consent
- <20 or >80 years of age
- Misuse of alcohol in the preceding 6 months,
- Episodes of hepatic decompensation leading to in-patient admissions during the previous month
- History or clinical signs of overt HE or severe sleep-wake disturbances
- On anti-HE treatment (lactulose, rifaximin, Hepa-Merz, Generaid, Bramino)
- History of significant head injury
- Neurological/psychiatric comorbidity needing medical treatment
- Taking neuroactive medication/medication known to affect sleep
- Travel across more than two time zones in the preceding 3 months
- Shift work in the preceding 5 years
- Contraindications for arterial puncture (negative collateral circulation test, wrist infection or vascular abnormalities).

Informed consent

Only subjects who have given a written and informed consent can participate partially or completely in the study. Both healthy subjects and patients will be given the same information by the principal investigator. The informed consent will be obtained after a personal meeting with the principal investigator. The consent must be based on both verbal and written explanation of the project, including the purpose of the study, risks and benefits as well as a presentation of the research group. The informed consent will be obtained in undisturbed surroundings with an invitation to bring a family member, friend or other companion. The subjects are not required to make the decision to participate at the time of the first meeting and further informational meetings can be arranged upon request at any time. The informed consent can be obtained after a

consideration period of up to one week after the first meeting. The subjects are given the following written materials at the informational meeting: Informed consent form, the brochure: “Forsøgspersoners rettigheder i et sundhedsvidenskabeligt forskningsprojekt” as well as written information for project participants (skriftlig deltagerinformation). The subjects can at any time withdraw their consent and require removal of already collected data.

Project plan

Day 1: Inclusion and randomization, introduction to the project, sleep questionnaires, ActiGraph, and informed consent.

Day 1-12: Home registration of sleep and ActiGraph monitoring. The subjects will be instructed to write a sleeping dairy.

Day 3 and 10: Experimental day. The subjects are fasting (for at least 6 hours) and will ingest either the AAC or glucose solution according to the randomization. At baseline and then with certain time intervals during the subsequent eight hours, the following is done:

- Karolinska Sleepiness Scale evaluation (every hour from intervention)
- Capillary ammonium measurement (every hour from intervention)
- Capillary adenosine measurement (every hour from intervention)
- Pupillometry (every second hour from intervention)
- Continuous reaction time assessment (every second hour from intervention)
- The psychometric portosystemic encephalopathy test (in the beginning and end of the day)
- Arterial blood measurement (one time at baseline)
- Generel eye investigation (one time at baseline)

Day 12: Final day where the subjects return the ActiGraph

AAC: The oral amino acid challenge consists of a flavored, 54 g amino acid mixture, mimicking the composition of the hemoglobin contained in 400 mL of blood [5]. The mixture is dispersed in 50-100 mL of water and ingested over a period of 10-15 minutes.

Isocaloric glucose solution: Flavored mixture or 72 g glucose dispersed in 50-100 mL of water and ingested over a period of 10-15 minutes.

Research biobank

The blood samples taken for plasma ammonium measurement will be analyzed directly and four samples of 2 ml each will be stored at -80 degrees Celsius for later analysis of other metabolites. The samples will be destroyed at the end of the project and will not be made available for other than the research group.

Outcomes

The primary outcome will be the difference between placebo and AAC in pupillary blue light reactivity measured as the pupillary area over time (area over the curve, see figure 1)

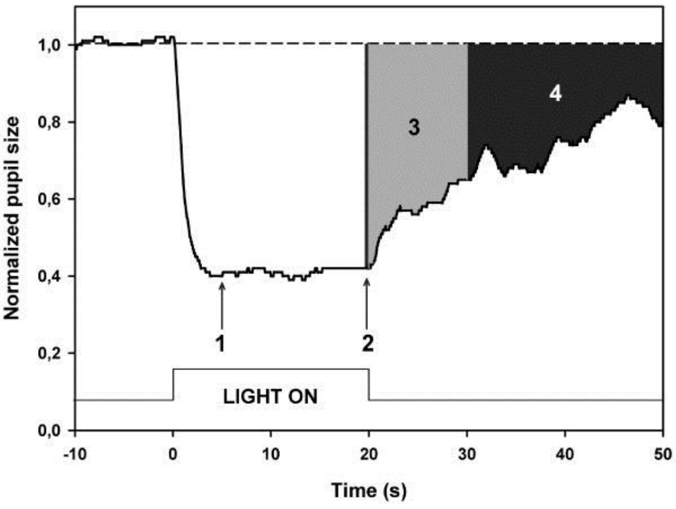

Figure 1: Example of a pupillometry measurement. The pupillary size is normalized in darkness (y=1) and the relative change in pupillary size is recorded over time in response to blue light exposure.

Secondly, we will study relations (regression analysis) between the blue light reactivity and the other measured variables: reaction time, arterial ammonia and sleep quality. Finally we will compare results between the group of patients and healthy controls (t-test).

Statistical analysis

We will perform paired t-tests on the primary outcome. A power analysis was performed with the "pwr" package for R (v.3.1.0) suggesting a sample size of 14.3 for paired t-tests. The following parameters where used:

- Effect size=0.8 (based on pilot studies in healthy subjects given AAC)
- Significance level=0.05
- Power=0.80

Risks, adverse events and inconveniences

The ingestion of the AAC has been found safe even in cirrhotic patients. It induces a transient and mild degree of sleepiness in healthy subjects and mimics a self-limiting and mild degree of hepatic encephalopathy in cirrhotic patients [14]. Since the AAC is simply a mixture of amino acids that normally is found in the diet, we do not expect any adverse effects. The sleepiness can be regarded as an inconvenience but we strongly advise against driving or operating machinery on the day of the experiment.

The only invasive part of our project is arterial blood sampling and capillary blood sampling. Both will be done according to general clinical guidelines. The patients will be informed of the risk of pain and small hematomas, and the quite unlikely (<1%) transient paresthesia.

The pupillometry itself is completely safe and without any risks, adverse events or inconveniences.

Confidentiality and data handling

All patient-related information and data obtained during the project will only be available to the principal investigator and in anonymized form to the research group. Both verbal and written information is protected under the act concerning the processing of personal data and the Danish health law (Lov om behandling af personoplysninger og sundhedsloven). The project is approved by the Danish Data Protection Agency.

Time schedule

September 2016: Start of patient inclusion

September 2017: End of patient inclusion

October 2017: Data analysis and manuscript preparation

Funding

The project will be funded through a pre-graduate scolarship on DKK 150.000 from Novo Nordisk Foundation. The subjects will not be offered financial compensation.

Ethical aspects and perspectives

The project will be conducted in accordance with the Helsinki Declaration. We consider the risk exposure of the subjects very limited and the potential inconvenience quite tolerable. The subjects can potentially benefit from the clinical assessment of sleep quality and the evaluation of ammonia sensitivity in respect to sleepiness and reaction time; however treatment of hepatic encephalopathy and related manifestations is not a part of this project. Indeed, treatment strategies for sleep disturbed liver failure patients are currently very limited and aim at correcting an underlying hepatic encephalopathy and the precipitating factors hereof. However, if the proposed hypothesis is confirmed, it indicates that these patients might benefit from cheap and non-invasive treatment with light-therapy. Potentially, our project's results could be translated to clinical practice immediately since these therapeutic measures already are readily available, although a randomized interventional study would be needed to justify this approach.

Description of the research group:

The research group, which has taken the initiative to this project, consists of: the principal investigator, Peter Nissen Bjerring, who is currently employed at the Gastro Unit, Hvidovre Hospital and Steffen Hamann, Department of Ophthalmology, Rigshospitalet - Glostrup. Peter

Nissen Bjerring has created the study design and will be responsible for recruitment and inclusion of subjects, as well as the practical arrangements involved. The development of and access to a valid pupillometry method has been provided by the neuroophthalmology research group, Rigshospitalet - Glostrup, led by Steffen Hamann. The present research group has access to all laboratory equipment needed and has routine as well as experience in handling cirrhotic patients, evaluate sleep disturbances and all methods mentioned. The research group has no financial conflicts of interests related to the project.

Dissemination plan:

2017: Presentation of preliminary data at the annual meeting of the American Association for the Study of Liver Diseases.

2017: Regardless of negative, positive or inconclusive results, publication of a manuscript in an international journal.

Reference List

1. Jepsen P, Vilstrup H, Sorensen HT. Alcoholic cirrhosis in Denmark - population-based incidence, prevalence, and hospitalization rates between 1988 and 2005: a descriptive cohort study. BMC Gastroenterol 2008;8:3.

2. Jepsen P, Vilstrup H, Andersen PK, Lash TL, Sorensen HT. Comorbidity and survival of Danish cirrhosis patients: A nationwide population-based cohort study. Hepatology 2008 Mar 26;48(1):214-220.

3. Montagnese S, De PC, De RM, Corrias M, Turco M, Merkel C, et al. Sleep-wake abnormalities in patients with cirrhosis. Hepatology 2013 Jun 6.

4. Shawcross DL, Olde Damink SW, Butterworth RF, Jalan R. Ammonia and hepatic encephalopathy: the more things change, the more they remain the same. Metab Brain Dis 2005 Sep;20(3):169-179.

5. Bersagliere A, Raduazzo ID, Nardi M, Schiff S, Gatta A, Amodio P, et al. Induced hyperammonemia may compromise the ability to generate restful sleep in patients with cirrhosis. Hepatology 2012 Mar;55(3):869-878.

6. Montagnese S, Middleton B, Mani AR, Skene DJ, Morgan MY. On the origin and the consequences of circadian abnormalities in patients with cirrhosis. Am J Gastroenterol 2010 Aug;105(8):1773-1781.

7. Schmoll C, Lascaratos G, Dhillon B, Skene D, Riha RL. The role of retinal regulation of sleep in health and disease. Sleep Med Rev 2011 Apr;15(2):107-113.

8. Hatori M, Panda S. The emerging roles of melanopsin in behavioral adaptation to light. Trends Mol Med 2010 Oct;16(10):435-446.

9. Park JC, Moura AL, Raza AS, Rhee DW, Kardon RH, Hood DC. Toward a clinical protocol for assessing rod, cone, and melanopsin contributions to the human pupil response. Invest Ophthalmol Vis Sci 2011 Aug;52(9):6624-6635.

10. Landolt HP. Sleep homeostasis: a role for adenosine in humans? Biochem Pharmacol 2008 Jun 1;75(11):2070-2079.

11. Basheer R, Porkka-Heiskanen T, Strecker RE, Thakkar MM, McCarley RW. Adenosine as a biological signal mediating sleepiness following prolonged wakefulness. Biol Signals Recept 2000 Nov;9(6):319-327

12. Boy C, Meyer PT, Kircheis G, Holschbach MH, Herzog H, Elmenhorst D, et al. Cerebral A1 adenosine receptors (A1AR) in liver cirrhosis. Eur J Nucl Med Mol Imaging 2008 Mar;35(3):589-597.

13. Bjerring PN, Hauerberg J, Jorgensen L, Frederiksen HJ, Tofteng F, Hansen BA, et al. Brain hypoxanthine concentration correlates to lactate/pyruvate ratio but not intracranial pressure in patients with acute liver failure. J Hepatol 2010 Dec;53(6):1054-1058.

14. Douglass A, Al MH, Record C. Amino acid challenge in patients with cirrhosis: a model for the assessment of treatments for hepatic encephalopathy. J Hepatol 2001 May;34(5):658-664.
