# Supplementary material for: The effect of induced hyperammonaemia on sleep and melanopsin-mediated pupillary light response in patients with liver cirrhosis: A single-blinded randomized crossover trial
Source: PLoS One. 2022 Sep 28;17(9):e0275067. doi: 10.1371/journal.pone.0275067 (PMC9518847; doi:10.1371/journal.pone.0275067)
Supplement: Attachment — Submitted filename: Response to Reviewers.docx [file pone.0275067.s006.docx]

**Response to reviewers**

**Journal Requirements**

Please review your reference list to ensure that it is complete and correct. If you have cited papers that have been retracted, please include the rationale for doing so in the manuscript text, or remove these references and replace them with relevant current references. Any changes to the reference list should be mentioned in the rebuttal letter that accompanies your revised manuscript. If you need to cite a retracted article, indicate the article’s retracted status in the References list and also include a citation and full reference for the retraction notice.

**Authors’ reply:** Thank you. We have ensured that the reference list is correct. We have not changed any current citations but have added an extra reference according to the reviewer's comments.

**Review Comments to the Author**

**Reviewer #2**:

There are no further comments from this reviewer - previous issues raised have been addressed adequately.

**Reviewer #3**:

**1.** The authors conducted an interesting, hypothesis-driven experiment with patients with a low-grade cirrhosis to test whether induced hyperammonemia acutely affect the pupillary response, as a surrogate measure of ipRGC function. There is a phase response curve to ipRGC; phase-shift effects response of ipRGC to the same intensity, spectrum, and duration of light will vary depending on the time of day. In addition, prior light history has been shown to affect the sensitivity of ipRGCs. Given the small sample size, it is not feasible to correct for multiple covariates. However, it will be useful to have additional information on the timing (both clock time and “circadian time” relative to the waketime of each subject) of measured pupillary response. It appears that the response was measured at hour 0, 2, and 4. Please include information on the clock/circadian time of these measures. Since ipRGC’s is most sensitive to light in the evening, the authors may consider including this in the discussion for a potential contributor to the lack of difference in the primary outcome.

**Authors’ reply:** Thank you for this important comment. We have now described it more thoroughly in the method section: “*The study assessments were performed at different time points during the inclusion period, as shown in Table 1. The experimental days ran from morning (8.00 am) until afternoon (3.00 pm) to reduce differences in the circadian rhythm between patients.”* And in the discussion, we have now included it as a potential contributor to the limited response: *“In addition, the daytime assessment may have affected the results, as ipRGCs are more sensitive to light stimulation in the evening.”*

**2.** Page 8. Pupilometer: What was the intensity of the blue light stimulation? Again, the response will depend on duration, intensity, and spectrum of the light.

**Authors’ reply:** Thank you for this comment. We have now inserted more detailed information about pupilometry: *“The duration of light stimulation was 20 seconds, and the illuminance level of both light colors was 100 lux.”*

**3**. Impaired sleep quality by which measure? PSQI? Please provide this information in the Table. (both baseline and after intervention)

**Authors’ reply:** Thank you for this comment. We measured the baseline self-assessed sleep quality by PSQI, which was not repeated after the intervention, as described in the method section. Besides, we measure the sleep quality during the inclusion period by Actigraph. We have now inserted the number of patients with high vs. low PSQI scores in table 1.

**4.** Child-Pugh Score an outdated measure of cirrhosis severity because of the subjective scoring. Can you provide MELD or MELD-Na score for the patients?

**Authors’ reply:** Unfortunately, the components for the MELD and further complications to the cirrhosis are not available in our data set. Therefore, we cannot change table 1. Anyhow, we consider the Child-Pugh Score a good and reliable instrument for evaluating the stage of liver disease.

**5**. The denominator of the Movement index should be TIB because TST does not include all of the “Time with movements during sleep”, which can be scored as wake during the sleep interval (therefore not included in TST). This likely won’t have a huge impact on the fragmentation index, but I wanted to make a note on it, disagreeing with the first reviewer.

**Authors’ reply:** Thank you for your comment on this. We have used the definitions provided by ActiGraph, the producer of the sleep evaluation watch. We have considered your note but have decided to keep the definitions suggested by Actigraph to promote reproducibility. Please find definitions in this reference: [ActiLife 6 Manual | ActiGraph (actigraphcorp.com)](https://actigraphcorp.com/support/manuals/actilife-6-manual/), page 62.

**6.** Discussion paragraph #3: How do you define superficial sleep? Would it be based on PSG study? I would use caution defining deep vs superficial sleep based on actigraphy measures. The subjects had less WASO and higher sleep efficiency after the experiment, regardless the intervention arm. Therefore, I would conclude that they were exhausted from a whole day of activities, which might have resulted in a better sleep.

**Authors’ reply:** Thank you for your comment. We have now changed the paragraph to clarify this: “*We would have expected that sleep after AAC decreased sleep efficiency because Bersagliere et al. [4] found superficial sleep after an equivalent AAC intervention in a similar population. However, differences in the study design could explain the divergence because Bersagliere et al. assessed sleep during a nap in the same afternoon with EEG analysis. Moreover, heavier sleep in our population could be due to exhaustion from a long experimental day rather than the intervention itself.”*
